# Perception of e-cigarette control policies and education in schools on increased legal knowledge, harm perception, susceptibility and e-cigarette use among students in Thailand: A cross-sectional classroom-based survey

**DOI:** 10.18332/tid/187840

**Published:** 2024-05-30

**Authors:** Chakkraphan Phetphum, Orawan Keeratisiroj, Atchara Prajongjeep

**Affiliations:** 1 Department of Community Health, Faculty of Public Health, Naresuan University, Phitsanulok, Thailand; 2 Tobacco Control Research Unit, Naresuan University, Phitsanulok, Thailand; 3 Department of Community Public Health, Sirindhorn College of Public Health, Phitsanulok, Thailand

**Keywords:** e-cigarettes, smoke-free policy, perceptions, susceptibility, Thailand

## Abstract

**INTRODUCTION:**

In Thailand, school smoke-free policies initially targeted cigarette smoking but now extend to include electronic cigarettes (e-cigarettes). Yet, the impact of this expansion on curbing e-cigarette use in schools is uncertain. This study seeks to explore how e-cigarette control policies and educational initiatives in Thai secondary schools influence students' legal knowledge, perception of harm, current e-cigarette use, and susceptibility to future use.

**METHODS:**

This cross-sectional survey was conducted in four regions of Thailand between May and August 2023, involving 6147 students selected through multistage sampling. Data collection utilized a self-reported online questionnaire in Thai, developed using Google Forms. For continuous outcomes, multiple linear regression models assessed relationships between school e-cigarette policy perceptions, covariates, knowledge of e-cigarette laws, and harm perception. Multiple logistic regression models analyzed the association between policy perceptions, covariates, and categorical outcomes: current e-cigarette use and susceptibility.

**RESULTS:**

Adjusting for covariates, a positive association was found between students' perceptions of e-cigarette policies and teachings and their knowledge of e-cigarette control laws (B=0.083, p<0.001) and harm perceptions (B=0.491, p<0.001). Additionally, these perceptions were protective against current e-cigarette use (AOR=0.970; 95% CI: 0.95–0.99, p=0.002) and susceptibility among non-users (AOR=0.962; 95% CI: 0.95–0.97; p<0.001).

**CONCLUSIONS:**

A heightened perception of e-cigarette policies and teachings in schools is associated with increased legal knowledge, heightened harm perception, and a lower likelihood of current or future e-cigarette use. This underscores the importance of enforcing the e-cigarette-free policy in schools to mitigate vaping susceptibility amid the ongoing youth e-cigarette epidemic.

## INTRODUCTION

The escalating use of electronic cigarettes (known as e-cigarettes) is recognized as a global public health concern^1^. A pivotal challenge lies in the marketing strategies directed at youth, characterized by modern designs and a strong emphasis on online promotional advertising^2,3^. This has rapidly increased popularity among young individuals^4^, including those who have never smoked traditional cigarettes before^5^. This group of young people is unnecessarily susceptible to health effects related to e-cigarette use^6^. The heightened susceptibility to associated health risks is, in part, attributed to the nicotine content in e-cigarettes, which is known to impede adolescent brain development and induce abnormal mood patterns^7-9^. Additionally, nicotine fosters addiction, increasing the likelihood of subsequent engagement in other substance use^10,11^.

As shown in previous research, perceived harm associated with e-cigarettes negatively correlates with both current youth e-cigarette use^12-15^ and susceptibility to e-cigarette use among non-users^15,16^. The school environment also plays a significant role in influencing both the current use of and susceptibility to e-cigarettes, incorporating factors such as smoking-free policies and regulations^17,18^, anti-e-cigarette campaigns^12,19^, and awareness of peer e-cigarette use^20,21^. Additionally, teachers play a crucial role in addressing these factors, particularly when they are well-informed about prevailing situations and e-cigarette use patterns among students, with their efficacy further heightened through adequate training in controlling students’ e-cigarette use^22,23^.

As youth devote a significant portion of their time to school activities, it becomes imperative to shield students from exposure to e-cigarettes in the school environment and provide school-based education to prevent the influence of deceptive tobacco industry advertising. Establishing effective nicotine and tobacco-free school policies is critical for achieving these goals^24^. In this context, teachers and school administrators are stakeholders and play vital roles in addressing issues related to e-cigarette use among youth^25^.

Smoke-free school policies have been widely implemented as a critical strategy to curb youth smoking rates. By prohibiting smoking on school grounds and establishing smoke-free environments, these policies reduce exposure to smoking cues and role modeling from adults, older peers, or friends who smoke within the school setting. Additionally, they promote comprehensive education, such as workshops on health risks or peer-to-peer education programs, and engaging activities that cultivate positive attitudes towards non-smoking. Ultimately, effective implementation of smoke-free school policies can make non-smoking the default choice for students and significantly lower youth smoking rates^26^. This strategy aligns with recent research indicating that students from schools with regular anti-smoking activities are half as likely to express an intention to smoke than students from schools with infrequent smoking activities^27^.

Although the landscape of youth tobacco use has rapidly shifted from traditional cigarettes to e-cigarettes^4^, schools remain a critical setting for intervention to prevent youth e-cigarette use. Nicotine and tobacco-free school policies are therefore being expanded in response to the changing context by providing an environment where children can learn about the dangers associated with cigarette and e-cigarette use, and policies that aim to reduce exposure to cigarettes and e-cigarettes and emphasize messages about the importance of avoiding these products. Additionally, schools provide an adequate opportunity because they reach many young people in a setting conducive to learning^28-30^.

Despite the ban on importing electronic cigarettes (e-cigarettes) into Thailand, e-cigarette use among youth is becoming a significant public health concern. With the rise of e-cigarettes among Thai youth, the Ministry of Education recommended that schools expand their smoke-free initiatives to include e-cigarette prevention^31^. Previous school-based surveys in Thailand found the prevalence of current e-cigarette use ranged from 3.3% to 3.7%^32,33^, with a higher prevalence among boys (5.5%) than girls (1.3%). However, the prevalence of ever using e-cigarettes among Thai teenagers has increased to 7.2%^29^. Thailand has implemented a comprehensive smoke-free school policy since 2005^31^ as a primary measure to curb youth smoking. The guidelines encompass the development of a smoke-free and nicotine-free school policy, establishing a smoke-free and nicotine-free environment, and integrating tobacco and nicotine education into the curriculum. This aligns with the World Health Organization’s recommendations for smoke-free school policies^24^. However, the implementation of these smoke-free school policies varies across schools due to voluntary compliance.

Previous studies indicate a low level of knowledge and awareness among Thai youth about the dangers of e-cigarette use, which is linked to current e-cigarette use^29-31^. This emphasizes the need for heightened attention to policy outcomes, particularly the quality of e-cigarette education assessed through students’ perceptions. This study examines youth perceptions and e-cigarette education provision in schools. The study also investigated the relationship between secondary school students’ perceptions of smoke-free policy and teaching in schools and their legal knowledge, perceived harm, current e-cigarette use, and susceptibility to e-cigarette use in Thailand.

## METHODS

This cross-sectional survey, approved by the Human Research Ethics Review Committee at Naresuan University (Certification No. COA No. 090/2023, IRB No. P3-0004/2566, certified on 12 April 2023), was conducted between May and August 2023.

### Sample and sampling

*Sample size estimation*

The sample size for this study was estimated using the infinite population proportion method. This method considers factors like the estimated prevalence of e-cigarette use among secondary school students in Thailand (p=0.072, derived from a previous study^29^), the desired margin of error (0.0072, representing 10% of the estimated proportion), the significance level (α=0.05), and a cluster effect of 1.2 to account for students being nested within schools. This resulted in a calculated sample size of 5942. To account for potential non-responses and incomplete data, a 10% buffer was added, bringing the final distribution of questionnaires to 6602 students.

*Sample selection*

**Figure 1:**
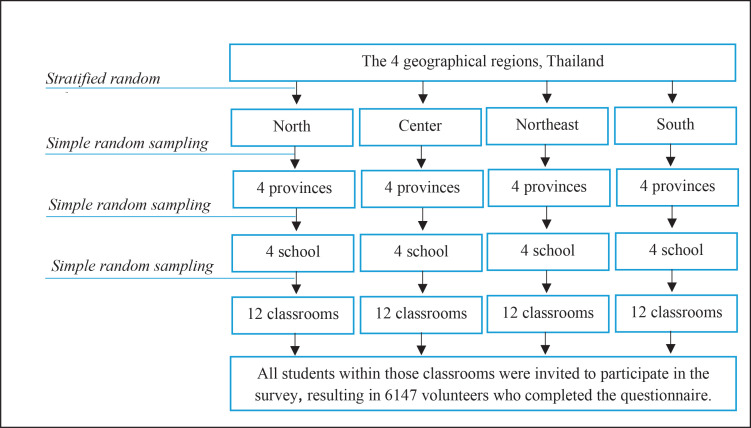
Flow chart of multi-stage sampling procedure, a cross-sectional classroom-based survey, Thailand, 2023 (N=6147)

A multistage sampling method was employed to select a representative sample of secondary school students aged 13–19 years in Thailand (Figure 1). Stratified random sampling was used to select six provinces from each of the four geographical regions (North, Center, Northeast, and South) to ensure the sample reflects the diverse geographical distribution of secondary schools (total of 16 provinces). The specific provinces selected were:

- North region: Chiang Mai, Tak, Phayao, Phitsanulok
- Central region: Phra Nakhon Si Ayutthaya, Chachoengsao, Samut Sakhon, Nakhon Pathom
- Northeast region: Khon Kaen, Ubon Ratchathani, Buriram, Sisaket
- South region: Songkhla, Surat Thani, Chumphon, Yala

Within each province, one school was randomly selected using simple random sampling. Twelve classrooms were then randomly chosen from each selected school using a simple random approach. Finally, all students within those classrooms were invited to participate in the survey. Out of the 6602 students invited, a total of 6147 secondary school students completed the survey, resulting in a high response rate of 93.11%. These students were included in the analysis for this study.

### Measures

A self-reported online questionnaire in the Thai language developed by the research team using Google Form was used for data collection. Content validity was assessed by three experts using the Index of Item-Objective Congruence (IOC). They evaluated the alignment between questionnaire items and the research’s operational definitions, rating each item as follows: 1 (clearly measures objective), -1 (not clearly measured), or 0 (unclear objective). The experts’ ratings were then used to calculate IOC scores for each item. The results indicated that IOC values for all questions ranged from 0.67 to 1.00, surpassing the acceptable threshold of 0.5.

Following this, the reliability of the validated questionnaire was tested by analyzing 35 secondary school students in Phitsanulok Province who were not part of the initial sample. Data obtained from the trial, which featured dichotomous response options, were used to compute confidence values using the Kuder-Richardson 20 (KR-20) formula. Likert scale questions were also analyzed for confidence values utilizing Cronbach’s alpha. The results revealed confidence values of 0.87 for knowledge regarding laws related to e-cigarette possession and use, 0.93 for harm perception associated with e-cigarette use, 0.91 for susceptibility to e-cigarette use, and 0.95 for perception of e-cigarette policies and teachings.

### Outcome variables

*Knowledge of e-cigarette-control laws*

This variable was measured with four questions (yes/no answers): 1) ‘Are e-cigarettes classified as a type of tobacco product according to the law?’; 2) ‘Are those who import e-cigarettes into the Kingdom of Thailand considered guilty?’; 3) ‘Are those in possession of e-cigarettes deemed guilty of an offense according to the law?’; and 4) ‘Are those who use e-cigarettes in public places considered guilty?’,

*Harm perceptions of e-cigarettes*

Harm perceptions of e-cigarettes were assessed by five Linkert scale questions (strongly agree, agree, neither agree nor disagree, disagree, and strongly disagree): 1) ‘Do you agree that people who use e-cigarettes are at risk of developing cardiovascular diseases?’; 2) ‘Do you agree that people who use e-cigarettes are more likely to develop severe pneumonia or EVALI?’; 3) ‘Do you agree that people who use e-cigarettes are exposed to toxins that adversely affect the brain?’; 4) ‘Do you agree that people who are exposed to secondhand e-cigarette vapors are at risk of developing bronchitis?’; and 5) ‘Do you agree that people who use e-cigarettes are likely to become addicted to nicotine?’.

*Current e-cigarette use*

Current e-cigarette use was measured with a single question: ‘Have you used e-cigarettes in the past 30 days?’. Those who answered ‘yes’ were asked additional questions assessing patterns of use: 1) ‘In the past 30 days, how many days have you used e-cigarettes?’ (from 1–2 days to use every day or all 30 days); 2) ‘How do you mostly use e-cigarettes?’ (using e-cigarette only, alternating with cigarettes, using cigarettes more than e-cigarettes, or using e-cigarettes interspersed with cigarettes with a preference for e-cigarettes); 3) ‘What type of e-cigarette do you use most often?’ (pod, mod, box, or other, along with a specification option); and 4) ‘Where do you get e-cigarettes and e-cigarette liquid from?’ (ordering online, purchasing from a friend or senior, or buying from a store/market in the community).

*Susceptibility to e-cigarette use*

Vaping susceptibility was considered only among non-users who responded ‘no’ to ‘Have you used e-cigarettes in the past 30 days?’ using Pierce’s validated measure^28,29^. Respondents were categorized as either susceptible or not susceptible based on their answers to three questions: 1) ‘Have you ever been curious about using an e-cigarette?’; 2) ‘Do you think that you will try an e-cigarette soon?’; and 3) ‘If one of your best friends was to offer you an e-cigarette, would you use it?’. The response options for all three questions included ‘definitely yes’, ‘most likely, yes’, ‘most likely, not’, and ‘definitely not’. Consistent with the findings of previous research^28,29^, respondents who answered anything other than ‘definitely not’ to any of the three questions were classified as susceptible to e-cigarette use. To prevent misclassification, respondents with a combination of ‘definitely not’ and missing information were classified as missing, as also observed in previous research.

### Independent variable

*Perceptions of e-cigarette policies and teachings in schools*

This variable was the primary independent variable of this research. It was measured with seven Linkert scale questions (strongly agree, agree, neither agree nor disagree, disagree, and strongly disagree): 1) ‘Do you agree that your school has a concrete policy to control e-cigarettes?’; 2) ‘Do you agree that teachers include content about the dangers of e-cigarettes in their teachings?’; 3) ‘Do you agree that teachers have educational materials on e-cigarettes for enhanced understanding?’; 4) ‘Do you agree that teachers use medical information about the effects of e-cigarettes in their teaching?’; 5) ‘Do you have confidence in the content that teachers teach about the dangers of e-cigarettes?’; 6) ‘Do you agree that the teaching content is up-to-date with new types of e-cigarettes?’; and 7) ‘Do you agree that the school regularly organizes anti-e-cigarette activities?’.

### Covariates

Previous research has established a link between sociodemographic and e-cigarette use among adolescents^30^. To account for the potential influence of these factors, this study considered the following sociodemographic variables: sex (male, female), current education level (middle school, high school), daily pocket money (specified in Tai Baht), and grade point average (GPA) (specified grade). Current smoking cigarettes were also regarded if they smoked a cigarette in the past 30 days (yes, no), and parental supervision of e-cigarette use was assessed by students’ perceptions of how often their parents supervise e-cigarette use (never or rarely, regularly). These variables were incorporated into the analysis to control their potential influence on the relationships between the independent and outcome variables. This approach helps isolate the specific effects of the independent variables on e-cigarette use while accounting for the influence of these sociodemographic factors.

### Data collection

After obtaining agreement from the schools to participate in the survey, the research team conducted a workshop via Zoom Cloud Meetings, which facilitated asynchronous meetings, to elucidate all the study’s details. These included the research objectives, the data collection process, and the potential implementation of the research findings. The workshop was attended by teachers responsible for tobacco control at the respective schools. These teachers were entrusted with administering the survey at their schools, acquiring verbal parental consent, and elucidating the study’s particulars to the students and their guardians. Verbal consent was sought to safeguard participant anonymity.

Once parental consent was secured, the researchers arranged Zoom cloud meetings with the targeted students. During these sessions, the students were briefed on how to complete the questionnaire and assured that their responses would remain confidential. They were also informed that the survey was anonymous and that teachers played no role in its execution. Furthermore, it was clarified that the data would be aggregated and presented in an aggregate manner only and that participants had the prerogative to participate or withdraw from the survey without providing a reason.

Following this explanation, students were allowed to pose any questions about the study and the data collection process. Subsequently, the researchers shared the link to the online questionnaire (Google Form) via the chat box in the Zoom cloud meetings and allotted up to 60 minutes for its completion.

### Statistical analysis

SPSS for Windows version 17.0 (SPSS, Inc., Chicago, IL, USA) was used to analyze the data. Descriptive statistics (frequency, percentage, mean, and standard deviation) were employed to summarize the characteristics of the data.

*Analysis of continuous outcomes*

Two separate multiple linear regression models were constructed to examine the relationships between perceptions of the school e-cigarette policy and teaching (independent variables), covariables, and the two continuous outcomes: knowledge of e-cigarette control laws and harm perception of e-cigarettes. All independent variables and covariables were entered into the model simultaneously (enter method) to assess their combined effect on the outcomes. Unstandardized regression coefficients (B) were used to evaluate the relative importance of each variable in explaining the variance in legal knowledge and harm perception. The R^2^ value indicated the proportion of variance in the outcome explained by the model.

*Analysis of categorical outcomes*

Two separate multiple logistic regression models were employed to analyze the relationships between perceptions of the school e-cigarette policy and teaching (independent variables), covariables, and the two categorical outcomes: current e-cigarette use and susceptibility to e-cigarette use. Like the previous models, all independent variables and covariables were entered simultaneously (enter method) to control their influence. The model fit was assessed using a Hosmer-Lemeshow goodness-of-fit. The analysis yielded adjusted odds ratios (AORs) with 95% confidence intervals (CIs) to estimate the association between the independent variable and each outcome while controlling for the other covariates.

Statistical significance was determined using a two-tailed p<0.05. The results of continuous outcomes are presented as unstandardized regression coefficients (B) along with their corresponding 95% confidence intervals and p-values. The results of categories outcomes are presented as adjusted odds ratios (AORs) along with their corresponding 95% confidence intervals and p-values.

## RESULTS

### Participant’s sociodemographic characteristics

The study included 6147 secondary school students who completed the questionnaire. Most participants (59.6%) were female, with similar proportions attending middle (53.8%) or high school (46.2%). The mean daily pocket money was 88.59 ± 43.35 Baht, and the mean GPA was 3.22 ± 0.59. Over the past 30 days, 29.6% of the sample reported smoking cigarettes, and 57.8% stated that their parents regularly advised against using e-cigarettes (Table 1).

**Table 1:** Student characteristics, a cross-sectional classroom-based survey, Thailand, 2023 (N=6147)

*Characteristics*	*n (%)*
**Sex** (N=6142)	
Male	2484 (40.4)
Female	3658 (59.6)
**Current education level** (N=6147)	
Middle school	3309 (53.8)
High school	2838 (46.2)
**Daily pocket money** (THB) (N=6116), mean ± SD	88.59 ± 43.35
**GPA** (N=5946), mean ± SD	3.22 ± 0.59
**Cigarette smoking in the past 30 days** (N=6147)	
No	5577 (90.7)
Yes	570 (9.3)
**Parenting against e-cigarettes** (N=6144)	
Never or rarely	2595 (42.2)
Regularly	3549 (57.8)
**Perceptions of school’s e-cigarette policies and teachings^a^** (N=6147), mean ± SD	27.06 ± 5.609
**Knowledge on e-cigarette-control laws^b^** (N=6147), mean ± SD	3.03 ± 1.34
**Harm perceptions of e-cigarettes^c^** (N=6142), mean ± SD	19.54 ± 4.33
**Current e-cigarette use** (N=6147)	
No	5555 (90.4)
Yes	592 (9.6)
**Susceptibility to e-cigarette use** (N=5519)	
No	4548 (82.4)
Yes	971 (17.6)

a Number of items=7, possible score range=7–35, score range 7–35.

b Number of items=4, possible score range=0–4, score range 0–4.

c Number of items=5, possible score range=5–25, score range 5–25. THB: 1000 Thai Baht about US$28.

### Perception of e-cigarette policies and teachings in schools

**Figure 2:**
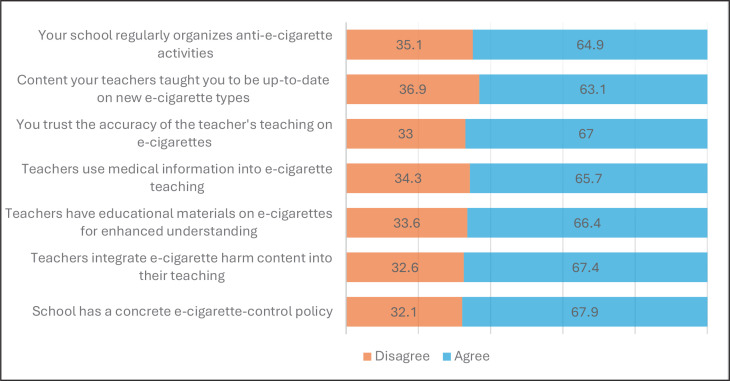
Perceptions of school e-cigarette policies and teachings classified by items, a cross-sectional classroom-based survey, Thailand, 2023 (N=6147)

According to Table 1, the average score for student perceptions of e-cigarette policies and teachings in schools was 27.06 ± 5.609, with a possible score range of 7 to 35. Among the survey items (Figure 2), the lowest level of agreement (63.1%) was observed regarding the perception that the content taught by teachers about e-cigarettes is up-to-date. Conversely, the highest level of agreement (67.9%) was noted for the statement indicating that the school has a concrete policy to control e-cigarettes.

### Legal knowledge and harm perceptions

**Figure 3:**
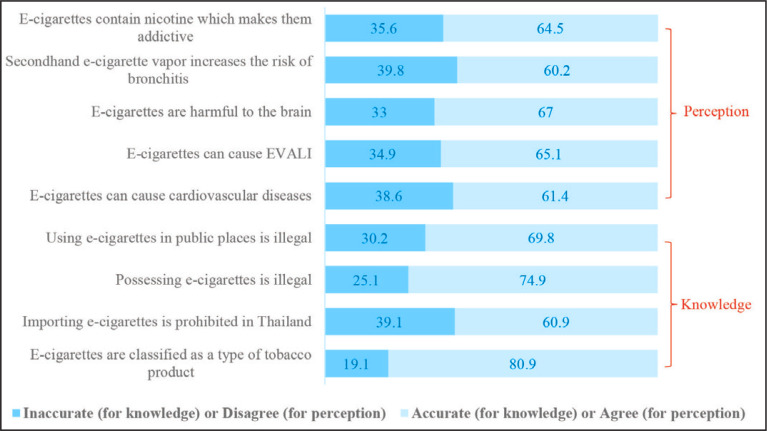
Legal knowledge, harm perceptions and susceptibility to e-cigarette use classified by item, a crosssectional classroom-based survey, Thailand, 2023 (N=6147)

The average score for student knowledge of e-cigarette control laws was 3.03 ± 1.34 out of a possible score of 4, as indicated in Table 1. Among the specific questions (Figure 3), students were least likely to answer correctly regarding the ban on importing e-cigarettes into Thailand (60.9%). Conversely, they demonstrated the highest level of knowledge regarding e-cigarettes being classified as a tobacco product (80.9%). Additionally, the average score for student harm perceptions of e-cigarettes was 19.54 ± 4.33 out of a possible score of 25. Among the statements, students showed the least agreement (60.2%) with the assertion that secondhand e-cigarette vapor increases the risk of bronchitis. Conversely, they expressed the highest level of agreement (67.0%) with the statement that e-cigarettes are harmful to the brain.

### Current e-cigarette uses and susceptibility to e-cigarette use

From the total sample, 9.6% (592 individuals) reported using e-cigarettes within the past 30 days. Among them, the majority (58.3%) were infrequent users, vaping only 1–2 days per month. Notably, nearly 70% (69.6%) of these users also smoked traditional cigarettes, indicating a pattern of dual use. Pods were the preferred device for 60.7% of users, followed by mods at 23.2%. The internet was the primary source for e-cigarette purchases for 66.7% of users, while 19.3% obtained them from peers or seniors.; 14.1% acquired these products from local retail outlets or flea markets. Among non-current users, 17.6% showed susceptibility to future e-cigarette use. This included 13.5% expressing curiosity, 14.4% considering trying soon, and 15.2% contemplating use if offered by a friend. These findings highlight a potential future user base.

### The relationship between the perception of school policies and teachings about e-cigarettes and legal knowledge, harm perception, current e-cigarette use, and susceptibility to e-cigarette use

In the forward enter multiple linear regression models, the analysis revealed a positive association between students’ perception of e-cigarette policies and teachings and their knowledge of e-cigarette control laws (B=0.083, p<0.001) and harm perceptions of e-cigarettes (B=0.491, p<0.001), controlling for all covariates (Table 2).

**Table 2:** Relationships between perceptions of school e-cigarette policies and teachings, and legal knowledge and harm perception, a cross-sectional classroom-based survey, Thailand, 2023 (N=6147)

*Variables*	*Legal knowledge ^a^ (N=6147)*			*Harm perception ^b^ (N=6142)*		
	*B*	*95% CI*	*p*	*B*	*95% CI*	*p*
**Sex**						
Female ®						
Male	-0.189	-0.255 – -0.124	<0.001*	-0.109	-0.284–0.065	0.221
**Current education level**						
Middle school ®						
High school	0.196	0.133–0.259	<0.001*	0.095	0.072–0.263	0.265
**Daily pocket money**	0.001	0.001–0.002	0.001*	0.003	0.001–0.005	0.004*
**Grade point average**	0.228	0.172–0.283	<0.001*	0.566	0.418–0.714	<0.001*
**Cigarette smoking in the past 30 days**						
No ®						
Yes	-0.001	-0.113–0.111	0.988	-0.308	-0.606 – -0.009	0.043*
**Parenting against e-cigarette use**						
Never or rarely ®						
Regularly	0.020	-0.043–0.083	0.538	0.151	-0.019–0.320	0.081
**Perceptions of e-cigarette policies and teachings in school**	0.083	0.077–0.089	<0.001*	0.491	0.476–0.506	<0.001*

a Constant= -0.076, R^2^ =0.160.

b Constant=4.145, R^2^=0.432. B: unstandardized coefficients.

* p<0.05. ® Reference categories.

Furthermore, in the forward enter multiple logistic regression models, students’ perceptions of e-cigarette policies and education emerged as a protective factor against current e-cigarette use (AOR=0.970, p=0.002) and susceptibility to using e-cigarettes among nonusers (AOR=0.962, p<0.001), controlling for relevant covariates (Table 3).

**Table 3:** Relationships between perceptions of school e-cigarette policies and teachings, and current e-cigarette use and susceptibility to e-cigarette use, a cross-sectional classroom-based survey, Thailand, 2023 (N=6147)

*Variables*	*Current e-cigarettes use ^a^ (N=6147)*			*Susceptibility to e-cigarette use ^b^ (N=5519)*		
	*AOR*	*95% CI*	*p*	*AOR*	*95% CI*	*p*
**Sex**						
Female ®	1			1		
Male	1.136	0.884–1.459	0.318	1.129	0.972–1.311	0.112
**Current education level**						
Middle school ®	1			1		
High school	1.092	0.857–1.391	0.476	0.828	0.715–0.959	0.012*
**Daily pocket money**	1.003	1.001–1.005	0.012*	0.999	0.997–1.001	0.422
**Grade point average**	0.452	0.372–0.550	<0.001*	0.680	0.600–0.770	<0.001*
**Cigarette smoking in the past 30 days**						
No	1			1		
Yes	59.471	46.190–76.570	<0.001*	3.366	2.401–4.719	<0.001*
**Parenting against e-cigarette use**						
Never or rarely ®	1			1		
Regularly	0.759	0.597–0.964	0.024*	0.821	0.710–0.950	0.008*
**Perceptions of e-cigarette policies and teachings in school**	0.970	0.950–0.989	0.002*	0.962	0.949–0.974	<0.001*

AOR: adjusted odds ratio.

a Hosmer-Lemeshow test: Step=1, χ^2^=10.827, df=8, sig=0.212.

b Hosmer-Lemeshow test: Step=1, χ^2^=14.340, df=8, sig=0.730.

* p<0.05. ® Reference categories.

## DISCUSSION

This research underscores the need to enhance smoke-free school policy in response to the current e-cigarette epidemic. The perception of e-cigarette-free school policies and education among secondary school students were found to be associated with increased knowledge of e-cigarette-control laws, heightened harm perceptions of e-cigarettes, and reduced susceptibility to e-cigarette use among non-current users after controlling for the covariates. These results contribute to the understanding of why students in schools with smoke-free and e-cigarette-free policies are less likely to engage in smoking and e-cigarette use^31-33^.

Despite the extensive use of the smoke-free school policy^33^ and evidence supporting its effectiveness in preventing youth e-cigarette use^12,17,18,21,24^, the mechanisms by which this policy achieves its goals remain poorly understood. A recent Australian study^23^ highlighted that secondary school teachers were aware of student e-cigarette use, leading to the implementation of e-cigarette policies, guidelines, and educational initiatives. However, the impact of these efforts on students’ perceptions of e-cigarettes remains unclear.

Apart from this, a study in the USA suggested that relying solely on a smoke-free school policy is insufficient to address e-cigarette use, but training teachers in designing and implementing educational programs is highlighted as essential^22^. However, this research did not investigate the impact of teacher training on students’ perceptions of the policies and their educational strategies.

Our study sheds light on the importance of student perceptions by evaluating how varied activities by the teachers and instructional methods influence their understanding of the smoke-free school policy. Students who held positive perceptions of school e-cigarette control policies and teachings demonstrated a stronger understanding of e-cigarette control laws and reported greater awareness of the potential harms of e-cigarettes. Furthermore, the analysis revealed a protective effect associated with these positive perceptions. Students with positive views were less likely to be current e-cigarette users and showed a lower susceptibility to future e-cigarette use. These findings highlight that effective nicotine- and tobacco-free school policies go beyond simply having regulations and health education. They must also cultivate positive student perceptions of these policies and the education surrounding e-cigarettes.

According to our study, the smoke-free school policy was introduced as a primary measure to prevent and control youth smoking and was subsequently extended to cover e-cigarettes following recommendations from the Ministry of Education^31^. Aligned with the WHO’s recommendation^24^, this policy encompasses establishing a smoke-free and nicotine-free school environment and integrating content on smoking and nicotine into teaching and learning. This approach fosters widespread positive awareness among secondary school students. In the present study, almost 7 out of 10 students perceived that their school’s concrete policy to control e-cigarettes was followed by their teachers incorporating content on the harm of e-cigarettes in their teaching. On the other hand, the aspect least positively perceived by students, compared to other items, was how up-to-date the content taught by teachers on new types of e-cigarettes. This suggests that teachers involved in teaching e-cigarettes in schools may experience delays in staying informed about new tobacco products despite their popularity among youth^22^. These findings underscore the necessity of addressing gaps in designing student instruction programs that are both timely and suitable. This challenge is particularly crucial given the rapid proliferation of emerging tobacco products and ongoing youth use.

Over 3 in 10 students did not perceive a concrete policy to control e-cigarettes in their school. This lack of awareness might be due, in part, to the voluntary nature of smoke-free school policies in Thailand. Each school implements these policies differently, leading to inconsistencies^33^. Furthermore, research suggests that policy alone is insufficient. Effective implementation relies on teachers’ knowledge and skills in designing e-cigarette prevention programs that align with policy goals^34^. Teacher training in school health initiatives has often been neglected^34^. In Thailand, there is a gap in knowledge regarding teacher training for e-cigarette prevention. This highlights the need to expedite teacher capacity building and development to address new tobacco products and industry tactics. The Ministry of Education should also consider raising minimum standards for smoke-free schools and e-cigarette policies, moving beyond voluntary measures. This comprehensive approach is crucial to effectively mitigate the proliferation of e-cigarettes in schools.

The national impact of school e-cigarette policies is unclear. This study sheds light on this gap by showing how different ways of implementing these policies can influence how high school students perceive them. Students who viewed their school’s e-cigarette control policies and teachings positively, demonstrated a stronger understanding of the laws and greater awareness of the harms associated with e-cigarettes. Additionally, these students were less likely to be current e-cigarette users and showed a lower risk of using them in the future. It is important to note that other factors need to be considered when examining the impact of school policies and teaching perceptions on students’ legal knowledge, understanding, risk perception, e-cigarette use, and future susceptibility. These factors include gender, academic performance, family financial situation, personal smoking history, and parents’ attitudes toward e-cigarettes. The study also revealed a knowledge gap, with one-third of students unaware of the illegality of importing e-cigarettes into Thailand. Similarly, accurate knowledge regarding vaping e-cigarettes in public was lacking. However, a majority of the students exhibited high levels of harm perception, although there was a poor understanding of the effects of secondhand e-cigarette vapor. To address these gaps, schools and teachers should design and enhance content while elevating teaching management specific to this issue.

### Limitations

This study acknowledges several limitations that offer valuable opportunities for future research. One limitation is the reliance on self-reported perceptions of school e-cigarette policies and educational programs among students. These perceptions may not always accurately reflect the actual implementation within each school, potentially influenced by factors such as student interests, biases towards teachers, or a desire to avoid e-cigarette education. Future research could incorporate objective data on how schools implement and enforce smoke-free and e-cigarette control policies to gain a more comprehensive understanding. Additionally, the study’s cross-sectional design restricts the ability to establish causal relationships. While it examines links between legal knowledge, harm perception, and receptivity to school policies, causality cannot be definitively determined. Future longitudinal research could provide clearer insights into these relationships over time. Furthermore, the variability in e-cigarette control policies and educational programs across schools presents another limitation. This study did not delve into the specifics of these programs, which can significantly influence student perceptions and behavior. Further research should explore the content and format of these programs to identify policy gaps and design effective educational interventions adaptable to the evolving e-cigarette landscape. Lastly, the administration of online surveys at schools might have influenced adolescent responses, potentially underestimating vaping behavior. Moreover, the participation of only 16 schools, likely those with existing policies, introduces a selection bias, limiting generalizability to the entire population of Thai secondary schools. Future research efforts would benefit from a larger and more representative sample to provide a more accurate picture of the influence of e-cigarette control policies and educational programs on students across Thailand.

## CONCLUSIONS

A school smoke-free policy has been implemented to control smoking and vaping e-cigarettes in schools, but the impact of the policy has been unclear. Our findings reveal that student perceptions of these policies vary significantly. Interestingly, students with more positive perceptions demonstrated greater knowledge of e-cigarette control laws, stronger awareness of the associated harms, and a lower likelihood of current or future e-cigarette use. These results highlight the importance of fostering positive perceptions of school e-cigarette policies and education. As the youth e-cigarette epidemic continues to escalate, schools should prioritize enforcing smoke-free policies and implementing effective educational programs to equip students with the knowledge and awareness to make informed decisions about e-cigarette use.

## Data Availability

The data supporting this research are available from the authors on reasonable request.
